# An Essay on the Laryngismus Stridulus, or Croup-Like Inspiration of Infants. To Which Are Appended, Illustrations of the General Principles of the Pathology of Nerves, and of the Functions and Diseases of the Par Vagum and Its Principal Branches

**Published:** 1836-07

**Authors:** 


### Art. V.

*An Essay on the Laryngismus Stridulus, or Croup-like Inspiration of Infants. To which are appended, Illustrations of the general Principles of the Pathology of Nerves, and of the Functions and Diseases of the Par Vagum and its principal Branches.* By Hugh Ley, M.D., Member of the Royal College of Physicians of London, Physician to the Westminster General Lying-in Hospital, Physician-Accoucheur to the Middlesex Hospital, and Lecturer on Midwifery at St. Bartholomew's Hospital. *Illustrated with Plates.—London,* 1836. 8vo. pp. 480.

We have frequently had reason to regret, in common with other practitioners, the various perplexities in which the disease which forms the subject of this essay was involved, and the utterly discrepant opinions that existed concerning it. We may venture indeed to assert that, in the lamentably long catalogue of maladies of which doubtful pathological and therapeutical opinions have been hitherto entertained, none more imperatively demanded impartial and deliberate investigation than the "croup-like inspiration of infants;" in the estimation of some distinguished medical authorities, an alarming and frequently fatal disease, requiring the prompt and decisive practice of free bleeding, "heroic" doses of calomel, and very slender diet;—in the opinion of others of equal eminence, a comparatively harmless, though tedious, complaint, rather aggravated than relieved by the treatment adverted to, and requiring for its cure the totally opposite practice of a tonic regimen, quinine, nourishing food, and country air. The very existence, indeed, of the disease, as separate and distinct from croup, has been a source of much dispute on the continent; and even here (constantly as we have opportunities of observing it,) it is not seldom mistaken for true croup by the inexperienced, and treated accordingly. Dr. Ley, therefore, is certainly not amenable to the charge of writing a book upon either an uninteresting or an exhausted subject.

As we are very desirous of taking as ample notice as possible of his opinions, we shall pass over the curious literary history of the "Laryngismus Stridulus" very briefly. It has been stated by different writers that this disease has but little attracted the notice of the profession. It appeared, however, to our author extremely improbable that a complaint dependent upon the physical agency of causes which have been always in operation, should have escaped the notice of practical medical writers; and, although it is true that the late Dr. John Clarke first fixed the attention of the profession to the subject, Dr. L. found, as he anticipated, that even from the earliest ages distinct notices of it were to be met with in medical works. The causes and intimate nature of this complaint, and the appropriate name whereby it should be designated, have been fruitful sources of contrary opinions; but the account of its characteristic features has been so uniform, that we are enabled at once to identify the disease as described by different writers under different titles. Until the beginning of the present century, this complaint appears to have been commonly described by the vague title of "asthma." In 1761, Dr. James Simpson published an inaugural dissertation, "de Asthmate Infantum Spasmodico." Dr. John Millar* noticed this disease in a separate publication, which has been the source of great controversy in this and other countries. He had seen much of the disease, and identifies it with that described by Simpson, whom he quotes. Dr. Ley finds little difficulty in recognizing, at least in the first stage of Millar's asthma, the complaint which Dr. J. Clarke termed "a peculiar species of convulsion in infant children." In the same year with Millar's book appeared, in the Philadelphia Gazette, Rush's brief account of this disease.† Underwood‡ briefly notices it; and Dr. Ley refers to Wichmann, as having given the best account of it of any foreign writer. Of late years this complaint has become more generally known, and many cases of it have been related in the medical journals. Dr. Ley refers particularly to the cases by Mr. Pretty;§ one by Dr. H. Davies,|| who records the dissection of a fatal case; and the observations of Mr. North, afterwards collected and remodelled in his work on the Convulsions of Infants.¶

"The two former writers, however, have been perhaps somewhat precipitate in their adoption of the speculative views of Dr. Clarke, as to the dependence of this disease upon vascular congestion within the head; whilst the last, who, when he first communicated his sentiments to the public, had never seen it terminate unfavorably, denied the connexion altogether, and combated with much success the Herculean attacks too indiscriminately made at once upon the disease and upon the constitution. Mr. North has since, in his 'Observations,' with the candour of an ingenuous mind, modified his first statement as to the universally slight character of this complaint. He acknowledges that it may be occasionally fatal, though much less so than has been generally believed; and he mentions one instance in which it destroyed the patient, and in which were discovered after death evidences of head affection; but he still denounces, in energetic terms, the heroic remedies so generally employed." (*Introduction*, p. xlviii.)

Gooch designated this disease by the term of "child crowing." Some interesting details respecting it were given by Mr. Hood, in the Edinburgh Journal of Medical Science for July, 1827. Dr. Marsh, in the Dublin Hospital Reports, vol. v., and Mr. Joy, in the Cyclopædia of Practical Medicine, have both contributed to our knowledge of the disease. In another part of the present

* Observations on the Asthma and Hooping-Cough. 1769.

† On the Spasmodic Asthma of Children, by B. Rush, M.D. London, 1770.

‡ On the Diseases of Children. Edit. 6. Vol. i. p. 51.

§ London Med. and Phys. Journal, vol. lv.

|| London Med. Repository, vol. xviii.

¶ Practical Observations on the Convulsions of Infants, p. 253. 1826.

number, we give an account of the complaint as noticed by the German physicians, with their peculiar views as to its causes. (See Part III. *On Thymic Asthma.*)

*History of the Disease.* Few affections of infants are more calculated to excite the attention and fears of the friends and medical attendant than the sonorous inspiration of "crowing children." This distressing malady, always formidable in appearance, and occasionally fatal in reality, consists essentially of a constriction of the glottis, which impedes the passage of air into the chest, even in the mildest cases, and sometimes suspends altogether for a time the respiratory function. The child has an interruption, more or less perfect, of its breathing, and, after vehement struggles, it at length succeeds in drawing in its breath with a shrill sound, like the peculiar inspiration of croup and hooping-cough, and arising from the same cause, a temporary diminution of the area of the rima glottidis. These attacks are paroxysmal, and vary in their frequency, severity, and duration. At first the intervals are considerable; but, in the progress of the complaint, the attacks become so frequent that the child scarcely rallies from one before another commences. In its earlier stages the attack is often very slight, and speedily and spontaneously yields, and terminates in a fit of crying succeeded by refreshing sleep. As the disease advances it increases in violence, and becomes unyielding in its character. In the earlier periods of the complaint, the attacks generally take place in the night, or after tranquil sleep; but, when it is firmly established, and sometimes from the commencement, they come on in the day from trifling or from no apparent causes.

After commenting upon the various and generally inappropriate terms by which the disease is designated, Dr. Ley adopts, as the least obnoxious to objection, the name of "Laryngismus Stridulus," given to it by Mason Good. It points out at once the seat of the affection and the characteristic symptom. The shrill sonorous inspiration, so characteristic of the complaint, marks very unequivocally its seat. It is evident that there is an unusual approximation of the sides of the glottis, as in croup or hooping-cough, in which there is also a close approximation of the sides of the rima glottidis.

"Where the closure of this chink is not perfect, the child struggles for its breath, the respiration is hurried, the countenance generally bluish or livid, the eyes staring, and each inspiration is attended with a crowing noise; where it is more complete, and this state at the commencement of the paroxysm, according to my observation, is much more frequent, the function of respiration is entirely suspended for a while, there is an effectual obstacle to the admission of air, the child makes vehement struggles, by some termed convulsive, to recover its breath; at varied intervals, from a few seconds up to a minute, or, upon some occasions, nearly two minutes, air is at length admitted through the glottis, now partially open, and this rush of air passing through a very narrow chink produces the peculiar sound. To these symptoms not unfrequently succeed a fit of coughing or crying, which terminates the scene; or, if the glottis be not even thus partially open, the child at the end of from two to three minutes, at the utmost, will die of asphyxia; pallid and exhausted, it falls lifeless upon the nurse's arm, and it is then that the child is generally said to have died in a fit." (P. 11.)

During the violent struggles for the recovery of the breath, all the muscles supplied by the respiratory system of nerves are thrown into violent action. The face is pale and cadaverous, and the external veins are turgid with highly carbonized blood. When the attack terminates fatally, it is not uncommon, immediately before death, to meet with true but generally slight convulsions. The peculiar contraction of the flexors of the thumb, fingers, wrists, ankles, and toes, is deemed by many writers an important, if not essential, mark of the disease. Dr. Ley thinks the frequency of this symptom has been over-rated by Clarke, North, and Marsh; he doubts, too, its convulsive nature, and regards these contractions as more nearly allied to feebleness of the extensors than spasmodic contraction of the flexors. This idea is opposed to the general opinion, but we have no doubt Dr. L.'s view of the subject is well founded.

"Another symptom which has escaped the notice of the greater number of writers upon this disease, but has not been overlooked by Mr. North, is the sound of mucus in the trachea. 'When the child wakes from its sleep, the breathing is for some moments unusually accelerated, and is accompanied by that kind of noise which an increased secretion of mucus in the air passages would produce.' This symptom is rarely entirely absent, but is particularly apt to occur either just before or for some time after a paroxysm. In other cases, however, I have known this symptom amongst the most prominent phenomena of the disease; lasting for weeks, sometimes with little interruption even for months, and attended from time to time by cough, so like hooping cough, as to deceive the most experienced. It is this symptom, indeed, which has led to the disease being confounded with the more severe forms of catarrh with increased secretion; and were it not for the freedom of the lining membrane of the fauces, nares, and frontal sinuses, from inflammation, would almost justify the terms 'catarrhe suffocant,' and 'catarrhus suffocativus,' by which this complaint has been known to some." (P. 15.)

Most writers are agreed in considering that there is a natural association of this disease with convulsions; but it may exist simple and uncombined, as described by Dr. Marsh and by Dr. Ley. The occasional association of this disease with the carpopedal contraction and general convulsions, led Dr. Clarke to consider that it was essentially connected with cerebral congestion and pressure; "but this opinion requires confirmation." Dr. Ley was at first inclined to yield to the high authority of Dr. Clarke, but more extensive and accurate observation led him to doubt, and ultimately to disbelieve, that such connexion is invariable or even general. He has been able to trace little in the symptoms of the disease, in the influence of remedies, or in the appearances upon dissection, to countenance, much less to establish, such relation. This is, in our opinion, a very important point. We are sure, from abundant observation, that Dr. Ley's experience has led him to a correct conclusion, and we are equally convinced of the mischief very often inflicted by those who pertinaciously adhere to, and who shape their practice by, the creed of Dr. Clarke.* Dr. Ley does not deny that the two maladies *occasionally* coexist.

*Causes of the Disease.—Age.* All writers agree in regarding this disease as one of infancy and childhood. Even those who allow the greatest latitude restrict it to within the ages of twelve or thirteen years; the most accurate observers confine its appearance to much more narrow limits. Professor Hamilton describes it "as peculiar to the age of cutting the deciduous teeth." Dr. Ley has seen it in one instance at four or five years of age, and in a second at six or seven;" but the great majority of cases unquestionably occur before the completion of the first dentition." This quite accords with our own experience; and we also agree with Dr. Ley, "that its all but exclusive appearance before the age of twelve or thirteen is probably to be referred to the size of the larynx at that time."

*Constitution.* A constitutional tendency to this complaint may occasionally be traced in whole families. Mr. Pretty states that three children of his own, and three in the family of a patient, were attacked in succession. Mr. North mentions an instance in which three children in the family were successively attacked, and Dr. H. Davies announces it as a general truth. Dr. Ley's experience amply confirms these statements, and we have recently had striking proofs of their accuracy.

*Climate and Season.* The disease, says Dr. Ley, is much under the influence of climate and season. In dry, elevated situations, and warm climates, it is little known; and hence the scanty information and erroneous views entertained respecting it by many of the German and some French writers, who frequently pronounce for the identity of a disease of which they know little or nothing, the asthma of Millar, with another with which they are very familiar, croup.† But, in the south and damper parts of France, the asthma of infants is well known, and is described by Baumes‡ as the "catarrhe suffocant nerveux."

* Dr. Merriman states, (edition of Underwood, p. 139,) "In two cases of the kind, the children died in the fits. They were both opened by Mr. Sweatman, a very skilful anatomist, but not the slightest appearance of cerebral affection could be discovered in either of them. The principal deranged structure discovered was a collection of small glandular swellings in the neck pressing upon the par vagum."

† Vide Albers Comment. de Tracheitide infantum, vulgo Croup vocata, p. 50; and also J. C. Albersii Comment. de Diagnosi Asthmatis Millari. Gottingæ, 1817.

‡ Des Convulsions dans l'Enfance. 1789. P. 365.

"It is probably to the change in the American climate, in consequence of the inhabited parts of that country being more cleared of its forests of timber, and its greater immunity, therefore, from diseases the result of fogs and damps emanating from the decay of vegetable matters, that the earlier American writers speak with so much confidence of this disease, whilst now they doubt its very existence; and this also probably accounts for the changes of opinion which Rush successively adopted in the intervals betwixt the publication of his letter to Millar in 1770, the publication of his first edition of Inquiries and Observations in 1794, and that of the second edition in 1805, with regard to this disease and its relation to croup." (P. 56.)

The constitutional tendency thus engendered by damp situations and seasons, is much aggravated by sudden vicissitudes of temperature; and hence the frequency of the disease in this country. The knowledge of this fact leads Dr. Ley to offer a few very proper remarks upon nurseries, which are generally but ill protected against such sudden alternations.

*Diet.* Indiscretions in diet notoriously aggravate the disposition to this disease.

*Scrofula.* Dr. Ley is inclined to believe that the causes just mentioned predispose to the disease, by establishing a disposition to scrofula.

"The similarity and even sameness of the circumstances which thus increase the tendency, at once, to the constriction of the glottis in infants and to scrofula, the great characteristic of which is glandular enlargement, would render it highly probable that there was some essential connexion between the two, even if there were no more direct and conclusive evidence upon the subject." (P. 59.)

Facts are given in support of this opinion.

*Exciting Causes.—Dentition.* The most judicious writers upon this disease concur in the assertion that it has much to do with the process of teething. The observation of Cruikshank, that the glands of the absorbents of the neck frequently "swell in children from inflammation of the gums and alveolar processes in teething, and recover after the teething is over," might have led to the suspicion that such swelling of the glands constitutes an intermediate link in the chain of events; and the experience and observation of Dr. Ley, and of others since the promulgation of his original views, confirm the suspicion. "Scarcely an instance has occurred to me, since my attention has been very strongly directed to the subject, in which there has not been the strongest foundation for the belief that either the glandulæ concatenatæ of the neck or the thoracic absorbent glands have become morbidly enlarged." Instructed by Dr. Ley, we have also paid particular attention to the subject, and, in confirmation of his statement, we may observe that, in one family, three children were attacked with this disease. In each the glands of the neck were enlarged, and in each the symptoms of the disease subsided as the glandular affection was removed by change of air and appropriate treatment. Dentition is analogous in its influence to that of any other cause of local irritation, common or specific, upon glands situated in the course of those lymphatics which lead from the part excited to the venous circulation in which they terminate.

"The inflammation of the gums, or alveolar processes, or enveloping membrane of the teeth, may produce morbid excitement of those glands in the neck, through which the lymphatic vessels from the teeth and gums are passing to their ultimate destination, and cause their enlargement even in the absence of all strumous taint; but still more certainly if such taint exist. This explains the fact noticed by Hamilton, that the complaint has 'appeared in the most robust as well as the most delicate infants,' and the statement of Etmuller, that it is a complaint of children 'plus minusve robustorum.'" (P. 62.)

*Inflamed and Ulcerated Scalp,* which is common in children from various causes, is generally attended with enlargement of the absorbent glands of the neck, and hence, again, may arise the croupy inspiration. Dr. Ley observes, that "the connexion of the inflamed and ulcerated scalp and enlarged glands has been rarely noticed, although it has not altogether escaped observation." We hope, and are inclined to believe, that he here under-rates the attention of practitioners to a very common chain of events. We apprehend that every medical practitioner, of even but moderate experience, is perfectly aware of the fact that in such instances there is almost always more or less glandular enlargement. It is a popular and even a professional opinion, that the sudden disappearance of eruptive ailments produces convulsions and other ailments in children, from the repulsion of acrid matter, rather than from the irritation of existing eruptions. This we have long doubted, and so has Dr. Ley, who has been, we believe, very correctly informed by Mr. Kelson, that reiterated experience had convinced him that, where convulsions were associated with such complaints, they were the result, and commonly in proportion to the extent, of the local excitement; that they had no reference whatever to the retrocession or repulsion of these eruptions; that they ceased as these appeared less angry or disappeared; and that, in such cases, the best mode of obviating the occurrence of convulsions, or other serious ailment, was to cure the eruption as speedily as possible. To this opinion we, with Dr. Ley, perfectly assent, "with a modification of the statement founded upon the necessity of attending to any internal cause, of which the eruption and the convulsions may have been the compound result, and of establishing some vicarious discharge, especially by the bowels, where the constitution has been long accustomed to the drain from an extensive surface, whether from the discharge of tinea capitis, crusta lactea, or excoriation behind the ears." As we know this is to a certain extent opposing a very popular impression and prejudice, we may observe that it is supported by the valuable authority of Bateman and Dewees. The latter* gives a caution, also given by Dr. Ley, that the diet should be restricted, and aperients administered, when the drain is suppressed. We know that the general, and as we believe imaginary, fears of the suppression of eruptive diseases in children, often reduces the practitioner to a mere spectator of a long-continued, and at least disagreeable and unsightly, malady, when he would, if he were released from the trammels of prejudice, adopt an active and efficient practice. In two cases detailed by Dr. Ley, it appears that the excitement arising from excoriations and acrid discharge behind the ears produced no "crowing" till glandular enlargement took place. When the crowing was violent, the increase of size of the glandulæ concatenatæ could be distinctly traced throughout their whole course: as by proper treatment their enlargement subsided, so did the functional derangement of the glottis.

"But the glands required some little time after the disappearance of the eruption completely to resume their original form and size, and, during this interval, a remnant, as it were, of the symptoms remained. It is upon the same principle that I would explain the fact, noticed by Mr. North, which has abundant foundation in fact, that, when this complaint arises from painful dentition, 'the symptoms do not vanish instantaneously, as if by magic, the moment a tooth starts through the gum. They pass off gradually.' In other words, after the gum and enveloping membrane of the tooth have been relieved from swelling and inflammation by the free use of the gum lance, some time is still requisite for the irritation and tumid state of the cervical glands to subside." (P. 64.)

*Head Affection.* Dr. Ley, aware of the practical mischief that has arisen from too readily adopting the opinions of Dr. John Clarke, or rather from pushing these opinions further than that very distinguished writer probably ever intended, argues this topic at much length, and very ably. Various practical authorities are referred to, in confirmation of the author's belief that, although vascular excitement of an inflammatory character within the cranium may occasionally produce the crowing inspiration, the weight of testimony is decidedly adverse to the conclusion that such relation is frequent, or that it is direct. "The pathological condition within the cranium may be the cause of the convulsions, and in some instances of the death of the child; but the breathlessness and crowing are the consequence of the enlargement of the glands."

"The head affection may be prior, and produce, by remote and indirect consequence, the laryngeal complaint; they may occur together, as mere coincidences, without any other association than the existence of a constitutional malady, which may be the common cause of both; the cerebral disturbance, again, may be the consequence of the frequent attacks

* On Children. Philadelphia, 1825. P. 316.

of breathlessness, almost amounting to asphyxia, which, by impeding the flow of blood through the lungs, causes accumulation within the cavities of the heart, and, subsequently, venous congestion within the cranium; and this may be followed by effusion of serum into the ventricles or between the membranes of the brain, and, occasionally and remotely, by inflammation and its ordinary consequences." (P. 81.)

Upon some occasions, Dr. Ley has had strong reason to suspect that simple bronchitis and disease of the lungs has, by contiguous irritation, caused enlargement of the bronchial glands, and thus rendered the original disease more intractable, and occasioned the crowing inspiration.

*Causes of the Paroxysm.* The symptoms of this disease are not constant: they recur at varied intervals, the intermissions being complete;

—"and the various other causes, which authors have enumerated amongst the occasional causes, are just such as excite the paroxysms. In considering these, I shall first notice those which are enumerated by other writers, and principally in the order in which they have noticed them, that it may not be imputed to me that I have allowed my observations to be warped by any preconception, which might be suspected to originate in the peculiarity of my views with respect to this malady." (P. 85.)

After mentioning the causes, of most frequent operation, enumerated by the best authorities, and which are apt to produce the paroxysms, Dr. Ley states that his own experience amply confirms the majority of the facts upon the subject which these writers have adduced: but, he continues,

"No one, however, as far as I know, has traced the mode of connexion of the predisposing and exciting causes of the disease with the phenomena which present themselves in its course; authors generally contenting themselves with alleging that it is a convulsive disease; that it arises from a variety of remote causes; and some, more minute, if not more accurate, in their speculations, referring it to an affection of the head, without, however, even an attempt to explain how it happens that this affection of the head should thus select one set of fibres, connected with this complicated chink, to the exclusion of all the others, although they all are close to each other, and derive their nervous influence from one common trunk." (P. 102.)

It results from the examination

—"that the causes of the paroxysm are such, as, by natural association without morbid influence, involuntarily close the glottis; that the breathlessness, which commonly precedes the sonorous inspiration, cannot, therefore, be from this closing of the rima glottidis, which, in such cases, is perfectly normal, but must arise from defective power in those agents, whose office it is to open that chink; and, lastly, that the crowing inspiration, which is nature's imperfect cure of the temporary suspension of breathing, arises from the chink being only partially open for the admission of air, and remaining so until some explosive expiration, such as screaming, crying, coughing, or belching, shall mechanically burst open the flood-gates, and perfect the recovery from the paroxysm." (P. 103.)

We have no doubt that Dr. Ley's claim to originality is perfectly well founded. For many years we have sought in vain for information upon the subject in the works of English and foreign writers, and have only found speculative opinions offered to explain the phænomena of this disease. We shall find ample reason, as we proceed, to come to the conclusion that Dr. Ley's views rest upon a much more solid basis.

*Pathology of the Disease.* Dr. Ley dedicates fifty pages of the work to the discussion of this part of the subject, and, as far as we know, the whole train of investigation, as well as the very important and well-ascertained results that flow from it, are entirely original. By nearly all commentators on this disease it has been believed that the temporary suspension of the breathing from a complete closing of the glottis, as well as the subsequent crowing inspiration from its imperfect opening, must have been the result, not of absolute or relative want of power in the opening muscles, but of excessive or anormal contraction of those which close the glottis. Hence the addition to its genuine name by a large majority of writers upon this complaint, of some adjunct or epithet which involves the postulate of its convulsive character. Dr. Ley very impartially examines the arguments that have been adduced to establish the convulsive character of this malady, and, among others, he very fairly criticises and corrects certain opinions that we entertained in common with others upon the subject, until taught better by his arguments and *facts;* and he succeeds in showing very clearly "that there is no sufficient evidence of the spasmodic character of the laryngismus stridulus."—"*It may be,* therefore, that the glottis is closed by the usual exertion of natural agents in the performance of some of the most ordinary functions, as swallowing, &c.; and *it may be,* that it remains partially or totally closed from some want of vigour in those antagonist powers which should again efficiently open that chink for the purposes of respiration; and to these conclusions an investigation of the remote causes, and their mode of operation, has already led me." Adopting, in the early part of his life, but never satisfied with, the current pathology of the disease, Dr. Ley was led to make minute and exact enquiries as to the class of constitution most liable to it, —of which the strumous is, beyond all comparison, the most frequent,—as well as into the causes which directly produced it. He found that the complaint arose from causes differing in situation and in essence; and he was driven, for the purpose of obtaining a more satisfactory explanation, to seek for some intermediate link in the chain of events common to them all, and of general, if not universal, occurrence, in combination with the laryngeal affection to which the symptom might be referred.

"The result of this inquiry has been to establish in my mind the conviction, that such common link, be the remote cause of this very peculiar symptom what it may, will, in the immense numerical majority of instances, be found to be enlargement of the thoracic or cervical absorbent glands. All the principal causes of the disease, as contradistinguished from the causes of the paroxysms, are capable of producing such enlargement, and experience confirms what a very general pathological connexion might have suggested; for I have found the relation between such enlargement and the crowing, when it has occurred, all but universal, and proved by observation during the life of the patient, or the examination of the body in fatal cases; and even in the very few instances in which it may have been difficult to detect the glandular affection, in consequence of the change of form in these glands from the compression of superjacent textures, or from their being within the thorax, the occurrence of similar symptoms might justify more than suspicion of its existence. 'Une maladie ne peut dépendre que d'une espèce de lésion.'" (P. 113.)

But how happens it that this fact has been so long overlooked? In the first place, the disease is "not commonly" fatal; secondly, examinations after death are generally carelessly conducted: those organs alone are examined which were previously suspected to have been in fault; and, thirdly, the very frequency and notoriety of enlarged bronchial or cervical absorbent glands has prevented them from being particularly noticed.

"But it is not enough thus to have traced such connexion: it is requisite that I should point out what is the nature of the relation which these two occurrences bear to each other,—to associate, in fact, the symptoms as the effect, with the pathological condition as their cause." (P. 114.)

This subject involves the results of minute anatomical research, physiological experiments, and pathological enquiries, and verifies the observation of Sir Charles Bell, that "it is the knowledge of the nerves of respiration distributed on the neck, throat, and thorax, that will enlighten the physician in distinguishing the symptoms of disease." The two clusters or chains of glands, subject, from a variety of causes, during infancy and childhood, to induration and enlargement, are in the immediate vicinity of organs of vital importance in the animal economy, and can scarcely fail, when enlarged, to exercise a pernicious influence upon parts with which they are then in contact. In children, absorbent glands are rarely sufficiently enlarged to encroach upon the area of the trachea, or much to interfere with the circulation of the blood; but they occasionally obstruct lymphatics in their course, and may impair materially the function of neighbouring nerves.* After arguing upon and satisfactorily establishing these points, Dr. Ley comes with apparent necessity to the conclusion that the phæ-

* In the Med.-Chir. Notes and Illustrations, Part I., by Mr. Fletcher, of Gloucester, the student will find many very interesting cases of "Spasm of the Glottis" from various causes.—Rev.

nomena of the disease must be accounted for by some influence which the enlarged glands exercise upon the respiratory nerves. In the Appendix, abundant and extremely interesting proofs are given that nerves may be affected in this manner. If there be much enlargement of the glands situated at the root of the lungs before and behind the bronchi, blending with others upon the arch of the aorta, not unfrequently between the origin of the carotids, and which are but continuations of those which follow the course of the trachea downwards, it is highly probable that the recurrent nerve, at its origin, may be subjected to their injurious influence, seeing that this nerve, upon the right side, turns round the subclavian artery, as it emerges from the innominata, behind which the nerve passes close to the origin of the carotid upon that side; whilst the left recurrent winds round the arch itself of the aorta, generally between the carotid and subclavian arteries, upon and about which these glands are situated. If, too, the deeper-seated chain of cervical glands are enlarged, they may affect the recurrent in its course; for they lie in the cellular tissue, which forms a cushion to that nerve. And now the curious and interesting question arises,—what would be the precise effect of such injurious influence, or positive pressure, upon the recurrent? This topic Dr. Ley argues at much length; briefly in the body of the work, but elaborately in the Appendix, where the reader will find the details upon which his statements rest, and may hence determine how far his inference is justified, that this disease, the spasmodic character of which has been conceded without examination, *is more allied to paralysis than to convulsive movement.* We regret that we cannot enter into the very interesting facts which Dr. Ley adduces in proof of this original position. We are satisfied that he has established it, and freely admit the instructive lesson we have received from him.

"This may appear a startling proposition to those whose habitual train of thought and mode of expression have referred to spasm as constituting the essence of this disease. But the prejudices of early education and impressions are not easy to eradicate; and belief, founded upon the presumed infallibility of the teachers whom we revere and the authorities which we respect, is apt to take irresistible hold of the mind, which yields to the seductions of indolence. It was long, therefore, before conviction was forced upon me by the reiterated cases which, since my attention has been more strongly directed to the subject, have presented themselves to my notice; and it may be probably the same with others. I thought '*more majorum*' that there was spasm from excess of nervous energy. The symptoms, however, if minutely examined, are not those of excitement of either the par vagum, or the recurrents; but they bear a striking analogy to those, which the experiments of physiologists have proved to be the result of an annihilation of the powers and attributes of those nerves, as where they have been tied or divided, or where a portion of them has been excised. When these respiratory nerves are excited, either by mechanical irritation with a pointed instrument, as in an experiment of Cruveilhier, or by inflammation in conformity with the pathological observations of Breschet, Autenrieth, and Gendrin, and as occurred in an interesting case recorded by Sir Astley Cooper, violent paroxysmal cough, like hooping-cough, has been, I believe, universally the result; but there are no attacks of breathlessness, amounting almost to asphyxia, as occur in this complaint. The other symptoms, also, show in this malady a state of paralysis in the other parts supplied from the same common trunk. So the transverse fibres, behind and connecting the rings of the trachea, losing their contractile power, the sputa accumulate in the air passages; hence the 'prodigious rattling in the upper part of the aspera arteria, resembling that sound which attends where there is phlegm that cannot be got up, scarce sensible when they are awake, but very great when they are asleep,' described by Dr. Molloy,—'that kind of noise which an increased secretion of the mucus in the air passages would produce,' noticed by Mr. North." (P. 124.)

Continuing the argument, which he fortifies on every side, Dr. Ley contends that, even if it were conceded that these glands acted by exciting these nerves, it would not only not explain, but would be inconsistent with the facts. "Excitement of these nerves would cause contraction of the muscles upon which the nerves are distributed; but this contraction would open the glottis, not close it; for the recurrents endow the opening, not the closing, muscles with contractile power." But the approximation of the sides of the glottis, thus produced by defective power of the opening muscles, may be either complete or partial. If complete, the child may be carried off by convulsion, or by asphyxia without convulsion. More commonly, however, the glottis becoming gradually but partially open, air rushes through the still contracted aperture, producing the sonorous inspiration so characteristic of this disease; and this commonly announces the partial recovery of the child. It is admitted that the laryngismus stridulus may occasionally depend upon a spasmodic affection of the closing muscles of the glottis;

—"but the general history of the predisposing and occasional causes of the disease, the exciting causes of the paroxysms, and the appearances upon dissection, all point to the conclusions, that such is rarely the case, and that an immense numerical majority of instances of this complaint are the result of a paralytic affection of the recurrent laryngeal nerves, produced by the compression of enlarged and indurated glands in their course." (P. 133.)

Several, and some very plausible objections may be brought forward against these novel views of Dr. Ley's. He investigates those that have yet been made, and alludes to others likely to be adduced; and he succeeds, in our opinion, in satisfactorily showing that the objections to his doctrine are more apparent than real. That the laryngismus stridulus of infants is very frequently mistaken by the public, and not seldom by medical practitioners, for other diseases, some of which more or less resemble and are analogous to it, and others from which it essentially differs, must be a well-known fact to all who are much consulted upon such subjects. The diagnosis of the disease is therefore an important topic, and, in the twenty pages Dr. Ley has devoted to its consideration, he clearly points out the distinction between it and other maladies with which it is so often confounded by the careless or inexperienced.

The *prognosis* in this disease has been a fertile source of variety of opinion.

"Simpson, Hamilton, Clarke, and Gervino consider it as a complaint which very materially implicates the safety of the patient; North, on the contrary, thinks it rarely fatal; whilst Millar, Rush, Underwood, Merriman, and Marsh, allege that it will generally yield to proper remedies. The means, however, recommended by Millar, Rush, and Underwood, although they resemble each other, differ from those suggested by Merriman; these, again, vary from the remedies advocated by Marsh; and Clarke adopts a practice different from each, and discountenanced by all." (P. 177.)

Dr. Ley's impression is, that, if this disease be treated early, and before the balance of circulation within the head has been much disturbed, that recovery may be expected in the majority of instances; and that those children have a much better chance of escaping who are not subjected to any severe discipline, than those who, in compliance with the prevailing doctrine with regard to this complaint, have been freely bled, had large doses of calomel, and such other remedies as the supposition of the invariable dependence of the disease upon cerebral turgescence or excitement has suggested. This doctrine we have always maintained, and more extended observation still further convinces us of its accuracy: and we must be allowed to express our surprise that, in spite of the host of facts arranged against it, the bleeding and heroic doses of calomel treatment is pertinaciously adhered to, from the unfounded apprehension that hydrocephalus must result from it, if this "protective" plan be not boldly enforced.

"The prognosis in individual cases of this disease is principally founded upon the age of the child, upon the frequency and severity of the attacks, upon the degree to which the glands are enlarged, upon the situation and cause of such enlargement, upon the extent to which the venous circulation within, or in its course from the head, is interrupted, and upon the existence or non-existence of the most formidable of all its combinations, that of arterial excitement and its consequences, including inflammation, serous effusion, and thickening of the membranes of the brain." (P. 180.)

*Treatment.* We perfectly agree with Dr. Ley, that the conclusions to which he leads us with regard to the nature of the disease are calculated to clear up much of the uncertainty and obscurity which has hitherto prevailed as to its treatment.* The primary

* Dr. Ley refers in a note to the evidence he has received, since the foregoing pages were printed, from different respectable practitioners whom he names, in confirmation of his pathological views of this disease.

object must be to ascertain if any of the glands so frequently alluded to are enlarged; to trace the cause of such enlargement, and to adapt our remedial measures to the cause. We must not infer the non-existence of glandular enlargements because we cannot detect them during life. Tumid glands even in the neck may escape detection; if within the thorax, they are not to be detected. "But, as similar diseased conditions commonly produce the same or similar results, we infer from the occurrence of the latter the existence of the former." But the pathological condition of these glands only establishes the liability to the attacks. The paroxysms generally require for their production the intermediate agency of some other event, which is to be considered as their exciting cause. It is therefore a second, but important, point in the treatment, to distinguish, and to prevent or to counteract, the operation of these causes of the paroxysms.

"As, moreover, the attack has been in some instances suddenly fatal by producing suffocation, the patient can never be considered as quite free from hazard, and it is necessary, therefore, so to treat each paroxysm as to shorten as much as possible its duration. This more particularly requires attention in those instances in which the breathing is completely suspended, for the restoration of which it is sufficient to procure, if practicable, even that imperfect inspiration which the crowing sound implies; for by this is insured, at least, the temporary safety of the child." (P. 194.)

The disposition to this disease is often a family peculiarity, but occasionally it is generated by the untoward agency of external circumstances, over which we have more or less control. Thus, in dry and elevated though cold situations, it is not frequent: the contrary obtains in low marshy situations. It is more frequent in crowded cities and large manufacturing towns than in the country. It is more prevalent in winter and spring than in summer or autumn. The admitted accuracy of these facts suggests appropriate care on the part of the friends, and appropriate counsel on the part of the practitioner, as far as climate, situation, and season, are concerned. "Warm clothing, too, is all important." Upon this subject Dr. Ley offers several remarks, which are not merely valuable in reference to the disease under consideration. We have often occasion to lament the folly and pertinacity with which children are exposed, half-naked in fact, but in perfectly fashionable attire, to our ever-varying climate. This is popularly called "hardening" the child. We regard it rather as an experiment to ascertain how much a child can bear without positive injury to its health. The mischiefs that hence arise are, unfortunately perhaps, not generally of immediate occurrence. If they were, the custom would, we suppose, be speedily abandoned: but, in spite of the "hardening" system, a delicate child does not "get on," and sooner or later scrofula appears in some of its forms, especially if there be any hereditary tendency to this, so called, and real "evil." It is very pertinently remarked, that, in dry and temperate weather, children ought not to be debarred from exercise in the open air, even if they have the mucous *râle* so frequently observed in this disease. There is no inflammation of the air-passages to be aggravated. In the ordinary language, there is merely an increased secretion of mucus; but Dr. Ley regards the symptom as dependent "upon accumulation of the ordinary secretion of the part in consequence of defective powers of the expelling apparatus, which is either enfeebled or paralysed; and upon this condition, weather has little other effect than as it has a tendency to enlarge or diminish the glandular tumours, upon the indirect influence of which that accumulation depends." The warm or tepid bath is a valuable auxiliary in the medical treatment; but let the practitioner remember that, if the child is frightened at the water, and cries or struggles violently, it ought not to be employed; for a paroxysm of the disease may thus be very probably produced. Cold bathing is objected to, and wisely, in this complaint. The *diet* of the child is of the utmost importance: it should, if possible, be confined to nature's food, the breast-milk, during the ordinary period of suckling. "The chances of preserving children who have a family proneness to this complaint will be exceedingly small if it be attempted to bring them up by hand." We know this to be correct: but insuperable difficulties may arise to prevent the child from being suckled. Here we must carefully regulate the quality and quantity of the child's food, and control, as well as we can, the prejudices of nurses. Asses' milk approaches nearest to the food provided by nature. If cows' milk is used, it should be diluted with about two-thirds of sweetened gruel. The grit or barley gruel, prepared barley, and farinaceous food, are more aperient than arrowroot, biscuit powder, flour, or rice. If, therefore, there be any tendency to constipation, the food should be selected from the former; if to diarrhœa, from the latter. Care must be taken, too, that the child is not too frequently fed, even upon proper food. The sucking bottle, with an artificial teat, is the best substitute for the breast. The earliest signs of disorder of the stomach and bowels demand attention, and it must be removed by appropriate change of food and proper medicines. Dr. Ley does not pretend to provide a remedy for scrofula; but, as a strumous taint has so much influence in producing the disease, he touches at some length upon the general management of children thus predisposed to it. Iodine may be employed with fair prospect of advantage in the cure of scrofulous tumours, and Dr. Ley's confidence in the doctrines he has advanced respecting the constriction of the glottis in children is much strengthened by the fact, that the two great remedies relied upon by Dr. Merriman are soda and burnt sponge, combined with aperients; though, from the obscurity which has hitherto hung over the pathology of this disease, Dr. M. had some difficulty in explaining their beneficial agency.

"Iodine or its salts may be employed both externally and internally.

The neck and upper part of the sternum may be rubbed twice daily with any simple unguent containing about one eighth of the hydriodate of potass, whilst for administration internally the solution of the hydriodate of potass, according to Magendie's formulary, constitutes as good a form as can be selected." (P. 232.)

*Dentition.* The most frequent local irritation that causes the disease is painful or difficult dentition, and here all writers strongly advise lancing the gums; and care must be taken that this little but important operation is efficiently performed. The gum, together with the membranous covering of the teeth, must be freely divided: the instrument should be felt to grate upon the tooth.

*Head Affection.* It is admitted that this complaint has occasionally an intimate connexion with morbid excitement within the cranium; and this relation may be one either of cause, coincidence, or consequence. The medical attendant, then, must be upon his guard, and appropriately modify his treatment to avert the threatened danger. In Dr. Ley he will find an excellent practical guide, who very carefully limits the extent to which debilitating measures can be safely carried in this complaint. If, after having subdued the convulsions, which are the great evidence of hydrocephalic excitement, the glands remain enlarged, and the "crowing" continues, "*pure country air* will do more to remove this secondary ailment than any remedies with which I am acquainted." For Dr. Ley's comments on the causes and treatment of the paroxysms we must refer to the work itself.

The Appendix, on the "Pathology of the Nerves," occupies nearly two hundred pages; and this we shall notice at a future opportunity. Its object is to establish the truth of those general principles connected with the pathology of nerves, which in the Essay Dr. Ley thought himself justified in propounding, and the truth of which further and more extensive enquiries have tended to establish and illustrate.

Every page of the work affords proof of the uncommon industry with which Dr. Ley has investigated the subject in all its bearings; and, in our opinion, the original views he entertains of the pathology of "Laryngismus Stridulus," which he derives from his own experience, and which he confirms at every point by references to various facts, physiological as well as pathological, contained in the numerous writers he has consulted, are perfectly correct. To these views we yield our perfect assent, not because we readily "catch at novelties," but because conviction is forced upon our minds. If we were disposed to be hypercritical, we might observe that occasional repetitions unnecessarily increase the size of the work, and that it would have been better to publish the Appendix in a separate form. By its length, the volume is of course rendered much more expensive, and thus we fear may spread less extensively among practitioners, who, without any exaggeration of its merits, will lose a very important and necessary lesson if they do not possess it.

